# Ultrastructural Organization and Metal Elemental Composition of the Mandibles in Two Ladybird Species

**DOI:** 10.3390/insects15060403

**Published:** 2024-05-31

**Authors:** Milos Sevarika, Roberto Romani

**Affiliations:** Department of Agricultural, Food and Environmental Sciences, University of Perugia, Borgo XX Giugno 74, 06121 Perugia, Italy; roberto.romani@unipg.it

**Keywords:** *Harmonia axyridis*, *Subcoccinella vigintiquatuorpunctata*, metal enrichment, TEM, SEM, EDX

## Abstract

**Simple Summary:**

Coccinellidae, commonly known as ladybirds, comprise a diverse group of insects with varied dietary preferences. Ladybirds typically feed on plants or prey on various insects, resulting in distinct morphological adaptations in their mandibles. Investigation of the ultrastructural organization of mandibles enables the identification of specific adaptations adopted by different species in response to their feeding modalities and preferences. In this study, we examined the ultrastructural organization of mandibles in two widely distributed and economically important ladybird species: the entomophagous *Harmonia axyridis* and the phytophagous *Subcoccinella vigintiquatuorpunctata*. Significant differences in mandible organization were observed between the two species. *H. axyridis* exhibited larger mandibles with a distinctive tooth pattern, whereas *S. vigintiquatuorpunctata* displayed multiple teeth on its apical region. Furthermore, variations in the metal content of the mandibles were identified, highlighting how dietary preferences influence mandible metal compositions.

**Abstract:**

The mandibles are among the most important appendages of insects’ mouthparts. Their morpho-functional organization is correlated with the variation in dietary preferences. In this study, we investigated the ultrastructural organization and metal composition of the mandibles of two ladybird species with different dietary habits: *Harmonia axyridis* (an entomophagous species) and *Subcoccinella vigintiquatuorpunctata* (a phytophagous species). The ultrastructural organization was studied using Scanning and Transmission Electron Microscopy, whereas the metal composition was investigated using Energy-Dispersive X-ray spectroscopy (EDX). Significant differences were observed in the general organization and metal enrichment pattern between the two species. The mandibles of *H. axyridis* are large and present a molar part with two teeth, with the apical one showing a bifid apex. In contrast, *S. vigintiquatuorpunctata* exhibited a molar region with several teeth on its apical part. The study revealed significant differences in metal content between the teeth and the prostheca of *H. axyridis*. Mn was the most abundant element in teeth, whereas Cl was more abundant in the prostheca. In the case of *S. vigintiquatuorpunctata*, Si was the most abundant element in the prostheca, while Mn was more present in the teeth. A comparison between the two species revealed that both teeth and prostheca showed significant variation in the elemental composition. These findings underscore the role of dietary preferences in shaping the structural and metal composition variations in the mandibles of these two ladybird species.

## 1. Introduction

Insects represent the largest and most diverse group of organisms, comprising about three-quarters of all described animal species. They play a crucial role in various ecological niches and play a key role in various ecosystems. With about 400,000 described species, Coleoptera (beetles) are the largest insect order [1,2]; although, it is estimated that the total number of all beetles may be much higher, approximately about 2 million species [3]. Within Coleoptera are ladybirds (Coccinellidae), with about 6000 described species [4].

The diversity of Coleoptera can be attributed to the availability of diverse food sources for insects. Inhabiting a wide range of habitats has driven these insects to evolve specific adaptations that promote diverse feeding preferences, feeding modalities, and the utilization various food sources [5,6]. Within Coccinellidae, there is a broad spectrum of dietary preferences, including mycophagy, pollinophagy, phytophagy, and entomophagy, with the latter two being the most numerous and significant [7,8].

The morphological characteristics of mouthparts evolved in line with species-specific feeding specializations, thereby enhancing their ability to effectively exploit diverse food sources. These adaptations are readily identified when two distant feeding modalities are compared, such as phytophagy and entomophagy. Phytophagous ladybirds (mainly belonging to Epilachninae) primarily feed by scraping the leaf surface and ingesting the plant juices, while avoiding ingestion of solid plant material. In contrast, entomophagous ladybirds feed by piercing and sucking their prey, rather than chewing them [9,10]. Sometimes, differences in mouthpart morphology may not always be readily discernible, as evidenced by the mandibles of anthophilous species when compared to entomophagous ones. This apparent lack of differences can be explained by the fact that entomophagous species use pollen as an alternative food source when their preferred prey is scarce [10].

Apart from evident morphological adaptations, the mandibles and other insect organs can be enriched by diverse inorganic compounds, which affect the biomechanical properties of the enriched structures. Frequently, these organs exhibit higher concentrations of transition metals (Cu, Fe, Mn, and Zn), resulting in increased hardness and stiffness [11,12,13,14]. Moreover, insect cuticles may undergo biomineralization, potentially enhancing the survival rate during aggressive behaviour between species or promote resistance to entomopathogenic fungi [15]. The metal composition varies between species. Until now, their presence has been documented in several insect groups: Diptera, Lepidoptera, Coleoptera, and Hymenoptera [14,16,17,18].

The metal composition and distribution vary not only between species but also between different positions on a specific organ or between different developmental stages [19]. These variations in metal accumulation correlate with the differing roles, hardness, and stiffness of associated substrates or materials.

*Harmonia axyridis* (Pallas), commonly known as the harlequin ladybird, is recognized as one of the most important ladybird species. Initially introduced as a biocontrol agent for various species, it has since become recognized as one of the most dangerous invasive species in the USA, Europe, and Africa. Besides preying on various insects, *H. axyridis* is also known to feed on fruits, vegetables, and pollen when primary food sources become scarce [20,21]. In contrast, *Subcoccinella vigintiquatuorpunctata* (L., 1758)*,* the 24-spot ladybird, is an important pest of *Medicago sativa* (L., 1753) [22,23]. When feeding upon host plant leaves, it produces characteristic damages by eating the lower layers of leaves’ epidermis and parenchyma, while the top layer remains in a membrane state.

In this study, we investigated the ultrastructural organization and metal accumulation pattern in the mandibles in these two important ladybird species. We hypothesized that feeding preferences could have an impact on the mandibles’ general morphology and metal accumulation pattern. We used Scanning and Transmission Electron Microscopy to reveal their ultrastructural organization, while Energy-Dispersive X-Ray Spectroscopy analysis (EDX) was employed to examine mandible metal enrichment.

## 2. Materials and Methods

### 2.1. Insects

Individuals of *H. axyridis* were obtained from a laboratory colony. Both larvae and adults were reared on an ad libitum diet of *Aphis fabae* Scopoli (Hemiptera: Aphididae), maintained on broad bean *Vicia faba* L. (Fabaceae). Insects were reared under controlled conditions (25 ± 1 °C, 70 ± 5% R.H. and L14:D10 photoperiod).

*Subcoccinella vigintiquatuorpunctata* individuals were collected from *M. sativa* plants in the Umbria region (Central Italy). Upon collection, insects were transferred to a rearing facility at the University of Perugia and maintained on *M. sativa* plants.

### 2.2. Scanning Electron Microscopy

Ten individuals of each species were immobilized via exposure to a low temperature (−20 °C) for two minutes, after which the mandibles were separated from the head using micro-scissors under a stereomicroscope. Then, the mandibles were dehydrated in a series of increasing ethanol concentrations (70, 80, 90, 95, and 99%, each step for 15 min), and then exposed to Hexamethyldisilazane (HDMS, Sigma-Aldrich, Dorset, UK) and allowed to dry under a ventilated hood at room temperature. Finally, the samples were mounted on aluminium stubs and gold-coated using a “Balzers Union SCD 040” unit (Balzers, Vaduz, Liechtenstein). Observations were carried out under a Field Emission Electron Scanning Microscopy (FE-SEM) LEO 1525 (Zeiss, Oberkochen, Germany).

### 2.3. EDX Analysis

Twenty mandibles, (10 mandibles for dorsal-side and 10 for ventral-side analysis), of each species were analysed. Samples were prepared according to the same protocol reported above for SEM investigations up to 99% ethanol dehydration. Subsequently, samples were air-dried at 35 °C in a temperature-controlled lab oven (M8-TB, Tecnovetro, Monza, Italy). Once ready, specimens were mounted on aluminium stubs and chromium sputtered using a Quorum 150T ES Sputter Coater (Quorum, Laughton, UK). Elemental compositions and chemical mapping were determined using a Bruker Quantax EDS (Bruker, Berlin, Germany), operated using the above-reported SEM. Samples were imaged and analysed using a beam energy of 20 keV, a suitable beam spot diameter for the particular magnification, and a working distance of 9 mm. Only cleaned specimens, i.e., those that did not show any surface contaminants were selected for the analysis. Areas of interest were first imaged, then the regions of interest were selected for EDS analysis. EDS microanalysis was performed in mapping mode, where each selected zone was scanned for five minutes. The zones were the mandibular teeth and the prostheca. Peaks of Mo overlapped with those of S. Moreover, due to software’s inability to discriminate between these two elements and to reliably determine their content, we discussed them together (Mo + S).

### 2.4. Light and Transmission Electron Microscopy

Five individuals of *H. axyridis* were immobilized via exposure to a low temperature (−20 °C). The mandibles were detached from the head using micro-scissors and forceps under a stereomicroscope. Once detached, the mandibles were transferred into a solution of 2% glutaraldehyde and 2.5% paraformaldehyde in 0.1 M cacodylate buffer + 5% sucrose, pH 7.2–7.3 at 4 °C overnight. Later, the specimens were rinsed in a buffer two times for 15 min, post-fixed in 1% OsO_4_ (osmium tetroxide) for 1 h at 4 °C, and washed in the buffer (two times for 15 min). Then, the mandibles were dehydrated in a series of graded ethanol (70, 80, 90, 95, and 99 for 10 min each step, except the last one that was repeated two times) and embedded in Epon-Araldite resin (Sigma-Aldrich, Dorset, UK) with propylene oxide as a bridging solvent. The ultrathin sections were taken using a diamond knife on a 2188 Ultratome Nova ultramicrotome (LKB, Stockholm, Sweden) and mounted on formvar-coated 50 mesh grids. The sections were stained with UranyLess (Electron Microscopy Science, Hatfield, UK) for 15 min and observed using a Philips EM 208 (Thermo Fischer Scientific Inc., Hillsboro, OR, USA). Digital pictures (1376 × 1032 pixels, 8-bit, uncompressed greyscale TIFF files) were obtained using a high-resolution digital camera MegaViewIII (SIS) (SIS, Muenster, Germany) connected to the TEM.

For light microscopy, thin sections (about 0.5 µm thickness) were taken using a diamond knife on a 2188 Ultratome Nova ultramicrotome (LKB, Stockholm, Sweden) and mounted on glass slides. The sections were stained with Toluidine Blue for 30 s and observed using an Olympus BX 53 (Olympus, Tokyo, Japan) microscope. Digital pictures were obtained using an Olympus XC50 (Olympus, Tokyo, Japan) digital camera mounted on the microscope.

### 2.5. Data Analysis

Mandible length measurements were performed in ImageJ [24]. EDX analysis was performed on individual teeth of ladybird mandibles. Given that investigated species differed in the number of teeth, values for individual teeth were merged, creating the new variable “teeth”. As a result, the metal composition of mandibles was compared between the two groups: teeth and prostheca.

Since the data did not follow a normal distribution, we conducted a non-parametric Wilcoxon test. We compared variation both intraspecifically (between the teeth and prostheca of each species) and interspecifically (each zone across species).

Pearson’s correlation was computed between elements to determine if there was a correlative effect within different groups (teeth and prostheca) for each species. The p-values of Pearson’s correlation were adjusted using the Holm method. All statistical analyses were performed in the R environment [25].

## 3. Results

### 3.1. Mandible Structure

**Figure 1:**
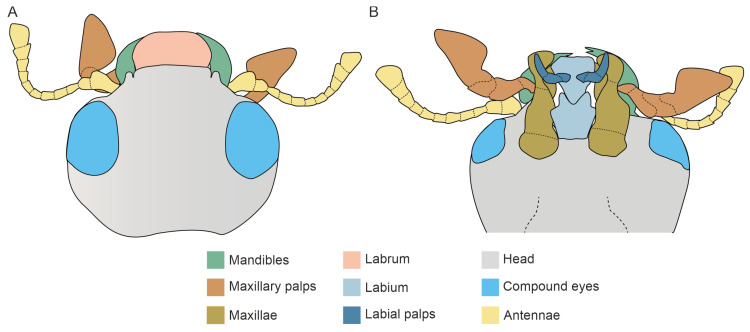
Schematic representation of *Harmonia axyridis* head. (**A**) dorsal view and (**B**) ventral view of the head. Different colours represent the different anatomical head appendages.

**Figure 2:**
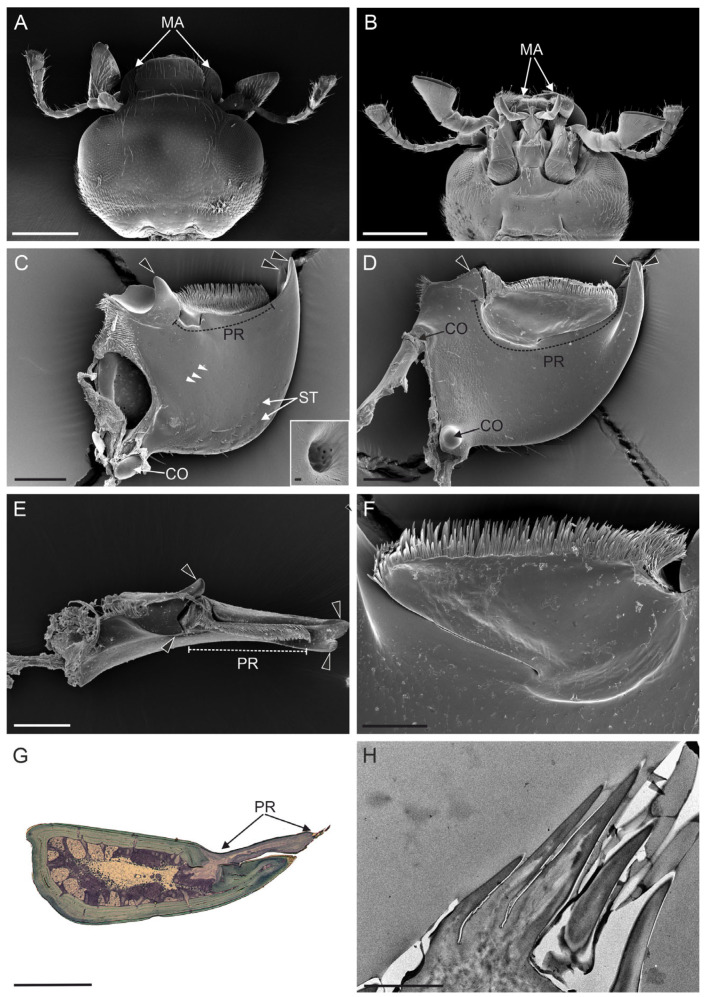
*Harmonia axyridis*. (**A**) SEM dorsal view of the head capsule showing the position of the mandibles (MA). (**B**) SEM ventral view of the head capsule showing the position of the mandibles (MA). (**C**) SEM image showing the mandible in dorsal view, with details of the molar teeth (arrowheads) positioned distally and proximally. The medial region is occupied by the prostheca (PR) and cuticular pores (white arrowheads). At the mandible base, a condyle (CO) and sensilla trichoidea (ST) are visible. Inset image: higher magnification image of a cuticular pore. (**D**) SEM image showing the mandible in ventral view. Besides the molar teeth (arrowheads), two basal condyles (CO) are shown. The PR completely occupies the region between the molar teeth. (**E**) SEM frontal view of the mandible, with evidence of the molar teeth (arrowheads) and PR. (**F**) SEM detail of the PR, showing the basal region connected with the mandible and the apical part characterised by abundant short setae. (**G**). Light microscopy picture showing a cross section of the mandible taken through the PR. It can be noted that the PR presents a thinner cuticle and is connected with the mandible only at the base. (**H**) TEM picture showing an oblique section of the apical part of the PR with the apical setae. The presence of sensory neurons was not recorded. Scale bars: (**A**,**B**) = 500 µm; (**C**–**E**,**G**) = 100 µm; (**F**) = 50 µm; (**H**) = 5 µm; and Inset = 200 nm.

Generally speaking, ladybirds presented chewing mouthparts characterized by a labrum, paired mandibles and maxillae, and a labium (Figure 1). The labrum was located dorsally, was narrower than the head capsule, and covering the other mouthparts. Below the labrum, two large, horn-shaped mandibles were visible, attached to the head through ventral and dorsal condyles (Figure 2A,B,D). In the two investigated species, mandibles had a different shape and also differed in size; on average, *H. axyridis* mandibles were 412 ± 6.22 µm (mean ± SE) long, while in *S. vigintiquatuorpunctata* the length was 339 ± 3 µm (mean ± SE).

**Figure 3:**
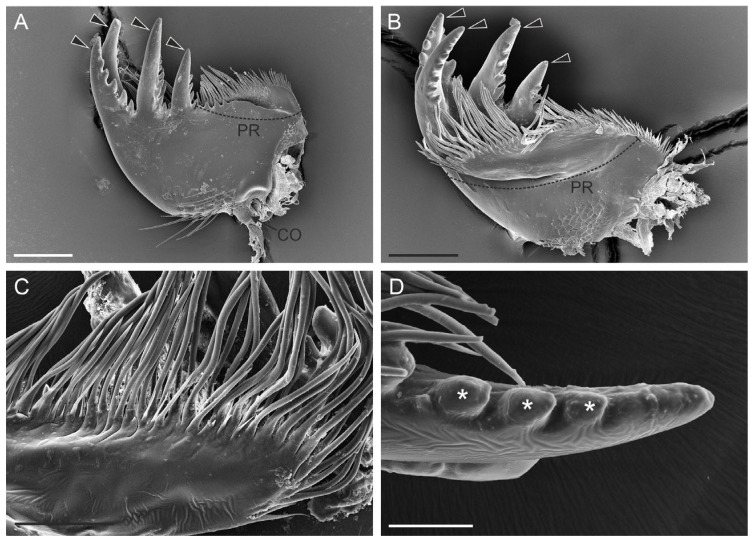
*Subcoccinella vigintiquatuorpunctata.* (**A**) SEM image showing the mandible in dorsal view. The half distal part of the molar region is occupied by the long, sharp cuticular teeth (arrowhead), while the half proximal part of the molar region is entirely occupied by the prostheca (PR), of which only the long setae can be observed. At the mandible base, a condyle (CO) can be observed. (**B**) SEM image showing the mandible in ventral view, with evidence of the mandible teeth (arrowhead) and the PR, which extends through the entire mandible molar region. (**C**) Close-up view of the setae that are present distally on the prostheca. (**D**) Close-up view of the internal edge of a mandibular tooth, with evidence of denticulate processes (asterisks). Scale bars: (**A**,**B**) = 100 µm; (**C**) = 50 µm; and (**D**) = 25 µm.

In both species, the cuticle generally had a smooth surface, except for the basal part, where sensilla trichoidea and numerous cuticular pores were found (Figure 2C and Figure 3A). The general structure of the mandibles revealed two well-defined regions: a distal incisor region and a proximal molar region. The distal incisor region in *H. axyridis* presented two sharp cuticular teeth, leading to a bifid apex without secondary denticulations (Figure 2C). Although the teeth were similar in length, the terminal part of each tooth was frequently broken or worn in some of the examined specimens.

The distal incisor region of *S. vigintiquatuorpunctata* exhibited four prominent cuticular teeth, three of which were positioned dorsally while one was located ventrally (Figure 3A,B). All these structures presented, on their innermost side (i.e., the side that, at rest, is facing the mouth opening), pronounced cuticular processes that gave a saw-like appearance to these elements (Figure 3D). *S. vigintiquatuorpunctata* had a distinct, rounded molar region without basal crushing teeth. Conversely, in *H. axyridis*, the molar region comprised two outward-pointing teeth, the dorsal tooth had a conical shape with a slight curvature, whereas the ventral tooth was rounded with a smooth margin. The prostheca, located between the molar and the incisor regions, extended over the ventral side of the mandible and was connected to its base (Figure 2D–G and Figure 3B). In both species, the prostheca was covered by several long, evenly distributed setae. The setae in *S. vigintiquatuorpunctata* were single-rowed and longer than in *H. axyridis,* which were distributed in several rows (Figure 2F and Figure 3C). Ultrastructural investigation in *H. axyridis* revealed a thinner cuticle at the prostheca base than the rest of the mandible; this could allow mobility during the feeding process. Moreover, the presence of sensory neurons associated with prostheca hair was not recorded (Figure 2F,H and Figure 3C).

### 3.2. Metal Distribution and Composition

**Figure 4:**
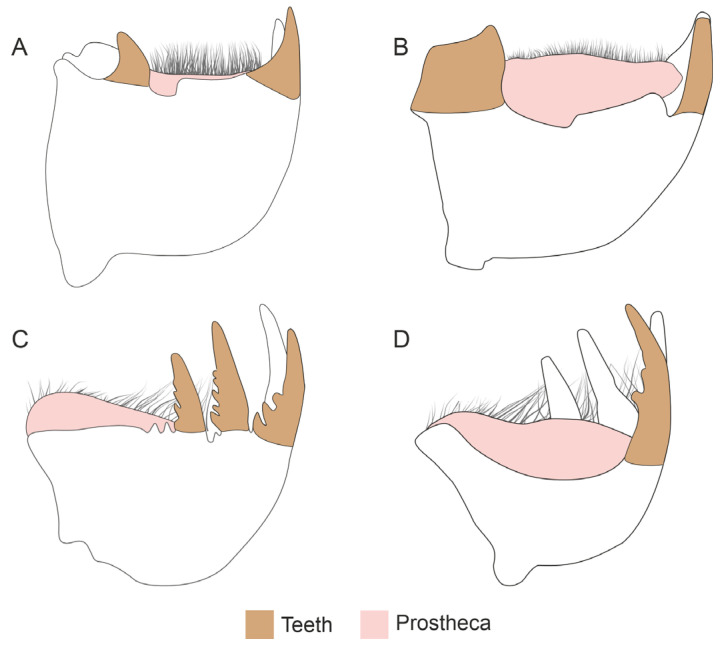
Schematic representation of the mandibles of *Harmonia axyridis* (**A**,**B**) and *Subcoccinella vigintiquatuorpunctata* (**C**,**D**) presented in dorsal (**A**,**C**) and ventral (**B**,**D**) views, respectively. Coloured areas indicate the zones being analysed for metal presence.

In both investigated species, we identified the presence of various metals in the mandibles at the level of the teeth and the prostheca, i.e., the zones that we selected for this analysis (Figure 4). In *H. axyridis*, the most abundant metals were Mn, Cl, and Mo + S. However, when comparing the presence of metals in specific zones, the most abundant elements were Mn (1.52%), K (0.29%), and Cl (0.24%) at the teeth level, whereas in the prostheca the most abundant were Cl (1.22%), Mn (1.12%), and Mo + S (0.58%) (Table 1, Figure S1).

When metal accumulation was compared at the intraspecific level, between the different mandible zones, significant variations were observed for Cl, Mn, P, Na, and Mo + S (*p* < 0.05, Table 1). In *H. axyridis,* these metals were accumulated more at the prostheca level, except for Mn, which was more abundant in the teeth. Fe was detected in limited amounts in *H. axyridis* mandibles. No significant variation between zones (prostheca vs. teeth) was recorded for Ca, Cu, Fe, Mg, K, Si, and Zn (*p* > 0.05).

Similarly to *H. axyridis,* in *S. vigintiquatuorpunctata,* the most abundant element was Mn, followed by Si and Cl. When comparing the elemental composition between teeth zones, the most abundant elements were Mn (2.2%), Cl (0.47%), and Mo + S (0.27%); in the prostheca, Si (1.38%), Mn (0.7%), and Cl (0.56%) were the most abundant elements (Table 1, Figure S2). Intraspecific differences in metal accumulation were observed for Mn, Si, Na, and Mo + S (*p* < 0.05).

In addition to the intraspecific variation in metal accumulation between zones, significant variations were recorded within zones between the two investigated species. In teeth, significant variations were found for Ca, Cl, Fe, Mn, K, P, Si, and Na. Most of these metals (Ca, Cl, Fe, Mn, P, Si, and Na) were more abundant in *S. vigintiquatuorpunctata* teeth, while only K was more present in *H. axyridis* (Table 2, Figure S3)

Differently from the teeth, the prostheca showed higher diversity in metals accumulation between the two species. Significant variations were observed for Cl, Mn, K (more abundant in *H. axyridis)*, Ca, Si, and Zn (higher accumulation in *S. vigintiquatuorpunctata*) (Table 2, Figure S4).

**Figure 5:**
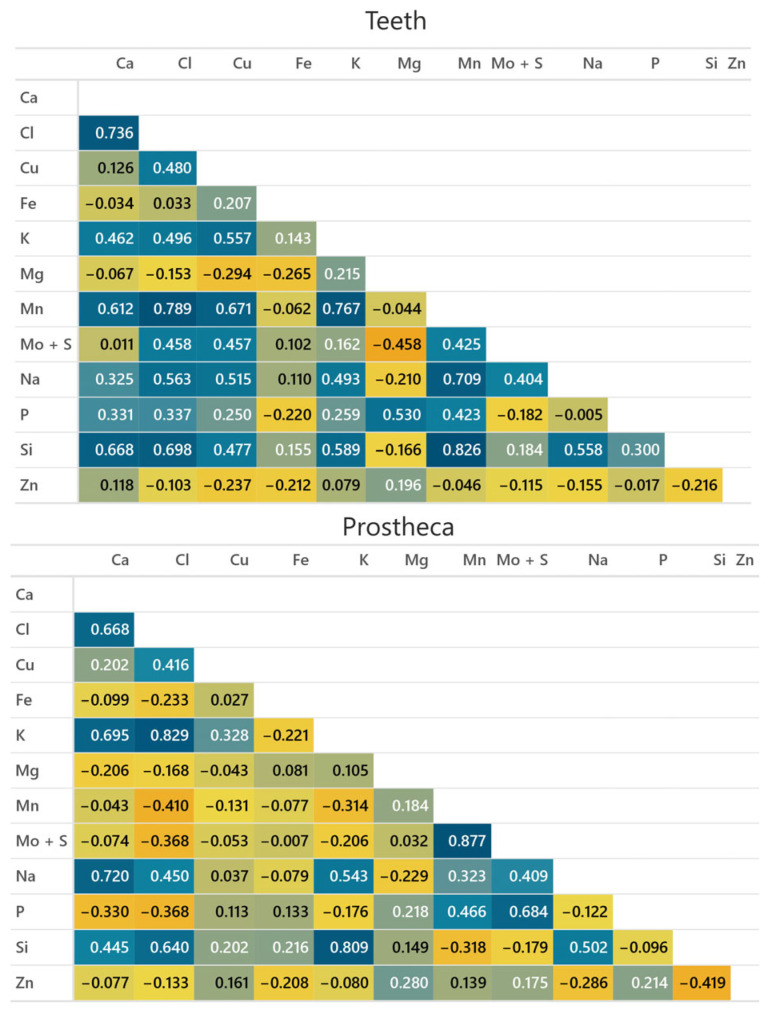
Pearson correlations (r) between elements found in the mandibles of *Harmonia axyridis.* The blue colour indicates a positive correlation, whereas the orange indicates a negative correlation.

**Figure 6:**
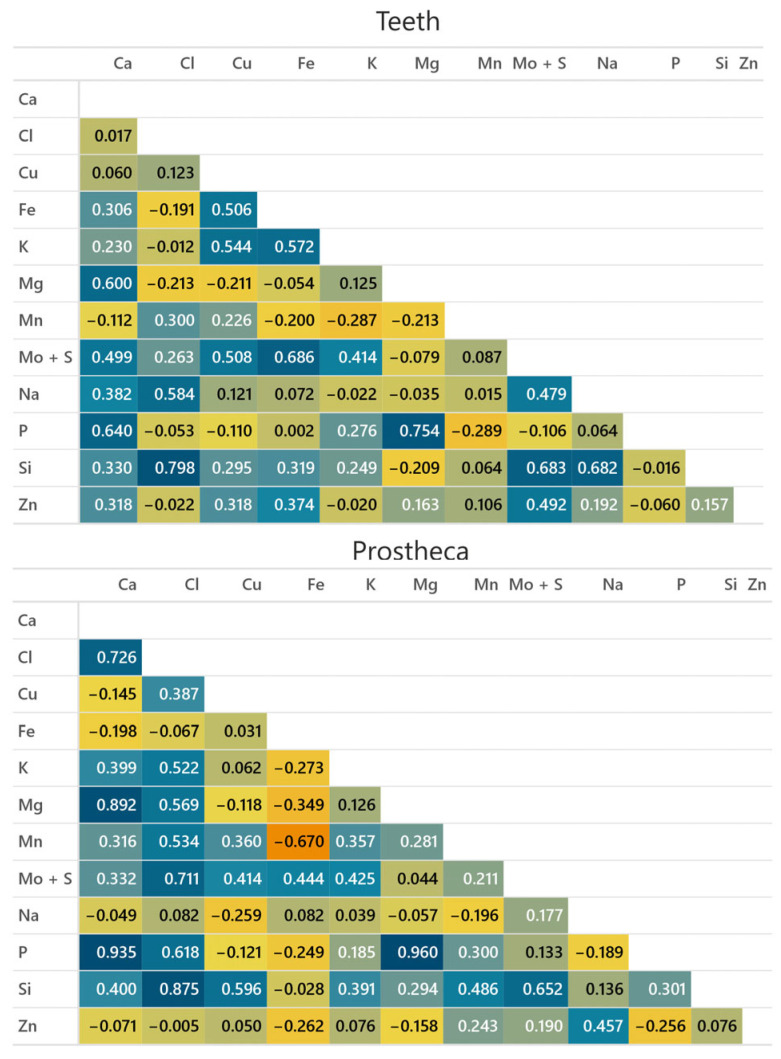
Pearson correlations (r) between elements found in the mandibles of *Subcoccinella vigintiquatuorpunctata.* The blue colour indicates a positive correlation, whereas the orange indicates a negative correlation.

Metals present in the cuticle of *H. axyridis* and *S. vigintiquatuorpunctata* showed close associations among themselves. In *H. axyridis* teeth, a statistically significant positive correlation was observed between Mn with Si, Cl, and K (*p* < 0.05). Moreover, a positive relationship was found between Cl and Ca (*p* < 0.05, Figure 5). In the prostheca of *H. axyridis,* we found a positive correlation between Cl and K and among Mn and Mo + S. In the case of *S. vigintiquatuorpunctata* teeth, an association in metal accumulation was found between Si and Mo + S, Cl, and Na; Mg and Ca and P; Fe and Mo + S; Ca and P; and Na and Cl (*p* < 0.05, Figure 6). Lastly, in the prostheca of *S. vigintiquatuorpunctata,* we detected a positive correlation between Si and Cl; Mg and Ca and P; and P and Ca.

## 4. Discussion

In the present study, we investigated the ultrastructural organization of the mandibles in adults of two ladybird species. We found significant differences in the mandible general organization and metal enrichment patterns between the entomophagous species *H. axyridis* and the phytophagous species *S. vigintiquatuorpunctata*. The mandible of *H. axyridis* featured a bifid apex with a two-toothed molar region. This particular mandibular organization has been reported for several carnivorous ladybird species and it is associated with its feeding modality. The entomophagous ladybirds feed by piercing and sucking their prey, rather than chewing it. Additionally, predatory coccinellids are known to have a unidentate tip, as observed in coccidophagous species such as *Chilocorus nigritus*, where it is adapted to lift the scale cover [9,26]. Conversely, the mandible of the phytophagous *S. vigintiquatuorpunctata* featured a multidentate mandible in the incisor region, used to scrape the leaf surface and extract the plant juices. Phytophagous ladybirds typically do not consume solid plant material, resulting in specific leaf damage, which can be used to confirm their presence [9,10,27,28,29].

Differences between the two species were observed in both the prostheca and the molar regions of the mandibles. Prostheca development varies between Coccinellidae. In most groups, it is attached along half the length of the mandible and it is equipped with a series of short or long setae. However, an exception to this rule was observed in Sticholotinae, where the prostheca extends along most of the mandible length and lacks setae [9,10]. In *H. axyridis,* the prostheca was well-developed with a series of short setae, indicating a mixed-feeding strategy. Indeed, *H. axyridis* is known to utilize pollen and fruits as an alternative food source when their primary hosts are scarce. In contrast, in other aphidophagous species such as *Coccinella transversoguttata* and *Hippodamia variegata* the setae were notably longer and distributed in a single row along the margin of the prostheca, similar to what was observed in *S. vigintiquatuorpunctata* [30]. When comparing prostheca development at the order level, it resulted to be highly variable. It is reported to be well-developed in anthophilous/pollinophagous and phytophagous species, whereas it is absent in specialized predatory coleopterans such as Staphylinidae (*Rudilus* spp.; *Stenus* spp.) [31,32]. The degree of prostheca development varies not only between families but also within a single family. In Chrysomelidae, for example, the prostheca was absent in Eumolpinae and Cryptocephalinae, reduced in Spulopyrinae, and well-developed in other groups [32]. Generally, the prostheca is a lobe-like mobile organ that plays a role in food gathering and concentrating pre-orally digested food originating from the prey or in scraping pollen grains and transferring them to the molar teeth [33]. Regarding the molar teeth, they were developed in *H. axyridis*, whereas in the case of *S. vigintiquatuorpunctata* they were transformed into a series of small teeth. Molar teeth are commonly observed in most Coccinellidae, except for Epilachninae, and are linked to feeding-related processes such as food grinding or pollen perforation. Molar structures were recorded in most insects, except Notoptera and Mantodea [5,9,34].

Mandibles and ovipositors are organs frequently subjected to various levels of mechanical stress [16]. Given their diverse function, frequently related to mechanical interactions with hard materials, these organs are exposed to different strain levels. Inadequate reinforcement of these important structures could lead to breakage, thus hindering insects from performing vital tasks. Reinforcement is typically achieved through metal accumulation, with transient metals such as Mn and Zn being frequently observed. In Coleoptera, many phytophagous species, as well as species attacking stored products, exhibit high Mn content in the mandibles, while wood-boring insects commonly contain both Zn and Mn [17,35]. Higher Zn presence was also recorded in Chrysomelidae, Ptinidae, Anobiidae, and Curculionidae [17,18,36]. However, in the coffee berry borer, *Hypothenemus hampei* [17,18,36], mandibles were not tested for Mn presence [36]. Among Curculionidae, in *Sitophilus granarius,* no Zn presence was recorded [17]. In other insect groups such as Lepidoptera, Orthoptera, Diptera, Hemiptera, and Hymenoptera, Zn is the predominant metal. Across all these groups, there is a tendency for single-element dominance, with the predominant element being conserved within a given family [18,37].

Although the mandibles of Hymenoptera have a higher quantity of Zn, their ovipositor is characterized by having Mn as the primary transitory metal. Species with Mn-enriched ovipositors are typically found to attack hosts concealed within hard substrates, to drill through woody materials, or to oviposit into hard-skinned fruits, as observed in the case of the *Drosophila* species equipped with strong, serrate ovipositor. This observation supports the hypothesis that Mn plays a role in hardening the insect cuticle [38,39].

Metal accumulation in the mandible is positively correlated with the parts that are subjected to more cuticular wear, such as the tip of the mandibular teeth, which process hard materials [40]. This correlation has been observed in several examples, particularly in leaf-cutting ants. These ants exhibit numerous serrations on their mandibles and show a gradual decrease in the metal content from the teeth edges toward the trailing part [37]. The decrease in metal content is found to be correlated with lower hardness and stiffness. This is in line with our observation, where we found higher metal accumulation at the teeth level compared to the prostheca.

Until now, studies on the functional role of Mn in the mandible are scarce. In *Atta sexdens*, the primary metal responsible for mandibles hardening was found to be Zn, while Mn content was minor [41]. However, even at low concentration (<2 wt.%), Mn is found to significantly contribute to mandible hardness and stiffness, exhibiting greater efficacy in hardening than Zinc [12]. This high efficiency of Mn could be related to the octahedral complexes formed by Mn ions, which enable up to six protein ligands, whereas only three protein ligands are expected in the case of Zn [12,42,43].

Indeed, the presence of Mn in the mandibles of coleopterans and several Hymenopterans, such as *Atta* ants, suggests that this metal provides necessary mechanical properties to the mandibles. In *S. vigintiquatuorpunctata* we observed a higher Mn amount compared to *H. axyridis*. Since leaves are often reinforced by silica and other metals, the insect cuticle needs to be hard enough to successfully cut through the leaf [44,45]. Due to the high silica content, insect mandibles are subject to wear [46,47]. The wear-off has a significant impact, especially on social insects such as leaf-cutting ants, which tend to change tasks in their colony when their mandibles become blunt. In fact, they switch from leaf-cutting role to carrying or other activities not related to the leaf-cutting process [48]. Since the primary food source of *H. axyridis* is aphids, which are soft-bodied insects, even a small reinforcement of the mandible is likely sufficient to successfully penetrate the aphid’s cuticle [49].

The mechanism by which Mn is acquired and accumulated in insects remains unclear. Previous phylogenetic analysis on Mn presence among Hymenoptera did not find an apparent association between life history traits and Mn accumulation, suggesting that Mn accumulation could be strongly influenced by phylogenetic factors [50]. However, some studies have suggested that diet could have a role in Mn accumulation. For example, when *Rhyzopertha dominica* was reared on a wheat germ diet, no Mn deposition was observed, but when 300 ppm of Mn was added to the diet, an increase in Mn content in the mandibles was observed [17]. Moreover, it has been hypothesized that the acquisition of one dominant transition metal could affect the acquisition of the second metal, i.e., acquisition of Mn could affect the deposition of Zn [50]. Schofield et al. [41,51] reported that the accumulation of Mn in ants takes place before Zn begins to accumulate; Zn accumulation begins abruptly about 10 h before eclosion and decreases as the adults age.

Apart from the high Mn concentration in the mandibles of coccinellids, we also observed a significant amount of Si. Si has been found in several insects and crustaceans. In crustaceans, it represents the primary metal, providing hardness and stiffness to the gnathobase teeth [52,53,54]. In the two investigated species, we found significant variations in Si content. *S. vigintiquatuorpunctata*, compared to *H. axyridis*, had significantly higher concentrations of Si in both the prostheca and teeth. This variation was particularly apparent in the prostheca. The differences in the Si content could be the result of the insect dietary preferences. Considering that *S. vigintiquatuorpunctata* is a polyphagous herbivore, mainly feeding on species from Poaceae, Caryophyllaceae, Solanaceae, Malvaceae, Polygonaceae, and Fabaceae, it is likely that Si was acquired from its food source [22,55,56]. The amount of Si varies among plant species, with the highest concentration found in Poales [57]. Consequently, it is possible that the prostheca is enhanced by Si, as it is responsible for transferring leaf pieces to the mouth. This is markedly different from *H. axyridis,* which does not have a Si-enriched prostheca, as it primarily feeds on soft-bodied prey.

Transition metals such as Zn or Mn are often present in complex distribution patterns, involving other elements. Zn is frequently associated with Cl, while Mn is often associated with Ca [11]. Halogen elements are commonly found to be correlated with transition metals [58]; as a result, they are linked to structural integrity [59]. However, it is not always the case that the presence of both transition and halogen metals will lead to an increase in hardness and stiffness of the structures [59]. Our correlation analysis detected a significantly positive correlation in the teeth of *H. axyridis* between Mn and Si, Cl, and P, while in the prostheca Mn had a significant correlation with S. Surprisingly, we did not detect a statistically significant correlation between Mn and other elements in the case of *S. vigintiquatuorpunctata.* Instead, significant associations were detected between Si and S, Cl, and Na.

## 5. Conclusions

In conclusion, we have investigated the ultrastructural organization of the mandibles of two ladybird species. We observed significant variations in the general organization of the phytophagous species *S. vigintiquatuorpunctata* and entomophagous species *H. axyridis*. The specific features present on their mandibles are likely associated with the feeding preference of each species. In addition, we found a significant intraspecific variation in metal accumulation in the mandibles, strongly supporting a morpho-functional specialization of these structures based on their feeding habit.

## Figures and Tables

**Table 1:** Metal accumulation in mandibles of *Harmonia axyridis* and *Subcoccinella vigintiquatuorpunctata*. The values represent mean % ± standard error.

Metal	*H. axyridis*		*S. vigintiquatuorpunctata*	
	Teeth	Prostheca	Teeth	Prostheca
Ca	0.07 ± 0.01	0.09 ± 0.02	0.19 ± 0.03	0.29 ± 0.06
Cl	0.24 ± 0.03	1.22 ± 0.16	0.47 ± 0.06	0.56 ± 0.06
Cu	0.23 ± 0.04	0.21 ± 0.04	0.2 ± 0.02	0.23 ± 0.02
Fe	0.01 ± 0.01	0 ± 0	0.03 ± 0.01	0.02 ± 0.01
Mg	0.03 ± 0.01	0.03 ± 0.01	0.03 ± 0.01	0.06 ± 0.02
Mn	1.52 ± 0.2	1.12 ± 0.14	2.2 ± 0.14	0.7 ± 0.09
Mo + S	0.17 ± 0.02	0.59 ± 0.08	0.27 ± 0.04	0.42 ± 0.07
P	0.03 ± 0.01	0.2 ± 0.04	0.13 ± 0.02	0.26 ± 0.07
K	0.29 ± 0.04	0.4 ± 0.09	0.04 ± 0.01	0.05 ± 0.01
Si	0.05 ± 0.02	0.05 ± 0.01	0.21 ± 0.03	1.38 ± 0.1
Na	0.07 ± 0.03	0.33 ± 0.1	0.14 ± 0.02	0.27 ± 0.03
Zn	0.03 ± 0.01	0.03 ± 0.01	0.06 ± 0.02	0.08 ± 0.02

**Table 2:** Statistical analysis of metal accumulation in *Harmonia axyridis* and *Subcoccinella vigintiquatuorpunctata.* The intraspecific variation section shows the *p* values when metal composition was compared between the teeth and prostheca of a specific species. The interspecific variation section shows comparisons in metal accumulation between the species for the specific mandible segment. The bold *p* values denote significant variations in metal accumulation (Wilcoxon test, α = 0.05).

Metal	Intraspecific Variation		Interspecific Variation	
	*H. axyridis*	*S. vigintiquatuorpunctata*	Teeth	Prostheca
Ca	0.563	0.106	**0.007**	**0.003**
Cl	**0.000**	0.261	**0.039**	**0.000**
Cu	0.967	0.144	0.468	0.614
Fe	0.688	0.564	**0.040**	0.071
Mg	0.731	0.497	0.983	0.342
Mn	**0.022**	**0.000**	**0.000**	**0.014**
Mo + S	**0.000**	**0.010**	0.198	0.064
P	**0.000**	0.106	**0.000**	0.840
K	0.711	0.099	**0.000**	**0.000**
Si	0.305	**0.000**	**0.000**	**0.000**
Na	**0.015**	**0.004**	**0.009**	0.538
Zn	0.611	0.099	0.307	**0.017**

## Data Availability

All data are available from the authors upon request.

## Supplementary Materials

The following supporting information can be downloaded at: https://www.mdpi.com/article/10.3390/insects15060403/s1, Figure S1. Metal accumulation in the mandibles of *H. axyridis*; Figure S2. Metal accumulation in the mandibles of *S. vigintiquatuorpunctata*; Figure S3. Differences in metal accumulation in the teeth of *H. axyridis* and *S. vigintiquatuorpunctata*; Figure S4. Differences in metal accumulation in the prostheca of *H. axyridis* and *S. vigintiquatuorpunctata*.
