# Supplementary material for: Ultrastructural Organization and Metal Elemental Composition of the Mandibles in Two Ladybird Species
Source: Insects. 2024 May 31;15(6):403. doi: 10.3390/insects15060403 (PMC11203409; doi:10.3390/insects15060403)
Supplement: Supplementary file 1 [file insects-15-00403-s001.zip › insects-3012498-supplementary.pdf]

Supplementary material for

Ultrastructural organization and metal elemental composition of the mandibles in two ladybird species

Milos Sevarika [1*], Roberto Romani [1]

[1]Department of Agricultural, Food and Environmental Sciences, University of Perugia, Borgo XX Giugno 74, 06121, Perugia, Italy;

*Correspondence: milos.sevarika@unipg.it

This PDF file includes:

Supplementary Figures 1 to 4

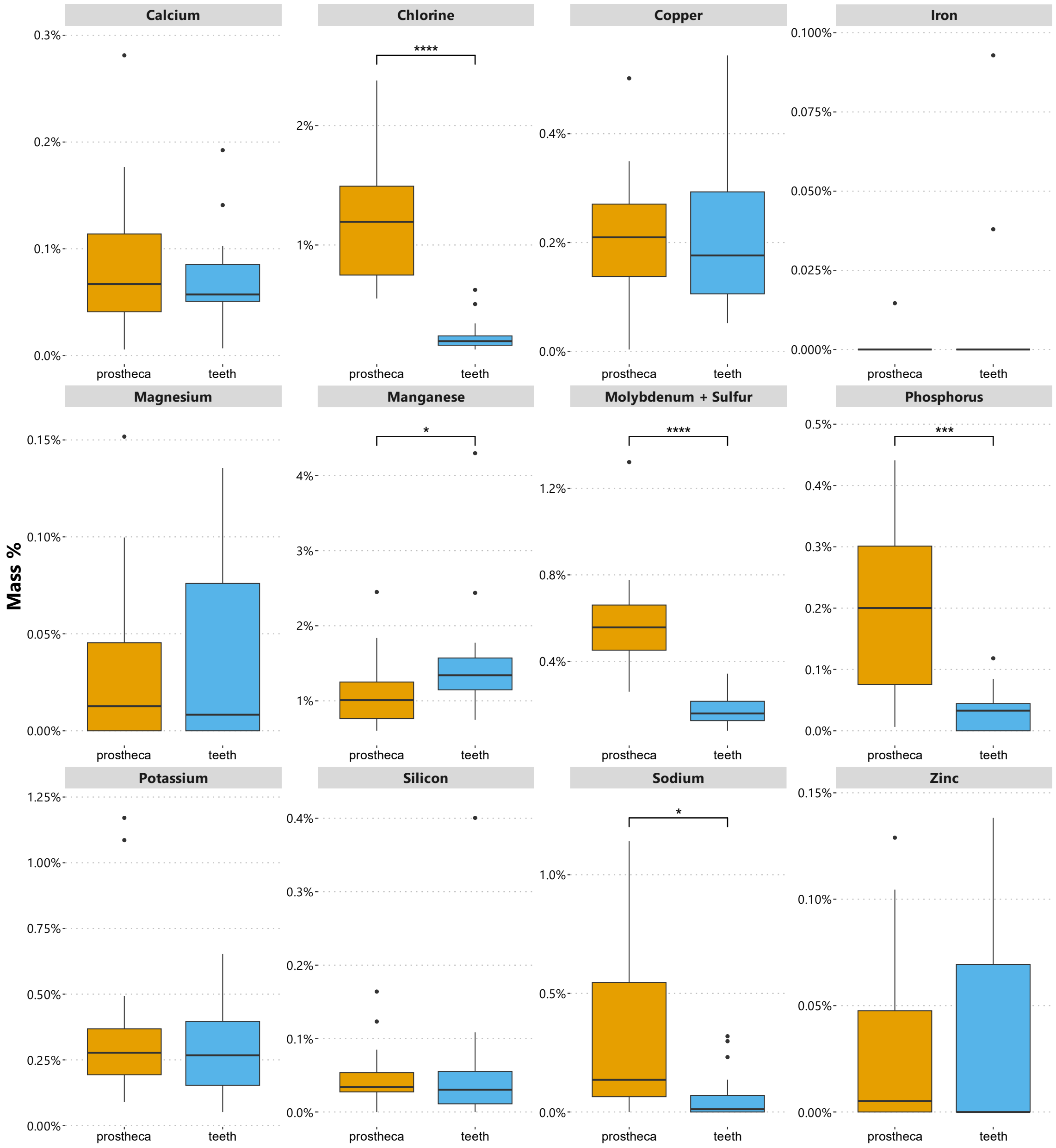

Figure S1. Metal accumulation in the mandibles of *Harmonia axyridis*. The box plots are showing metal abundance in the prostheca and teeth of *H. axyridis*. Center line in each box plots represent the mean value, with standard error bars. Asterisks indicate significant variation in metal accumulation between prostheca and teeth (Wilcoxon test, α=0.05).

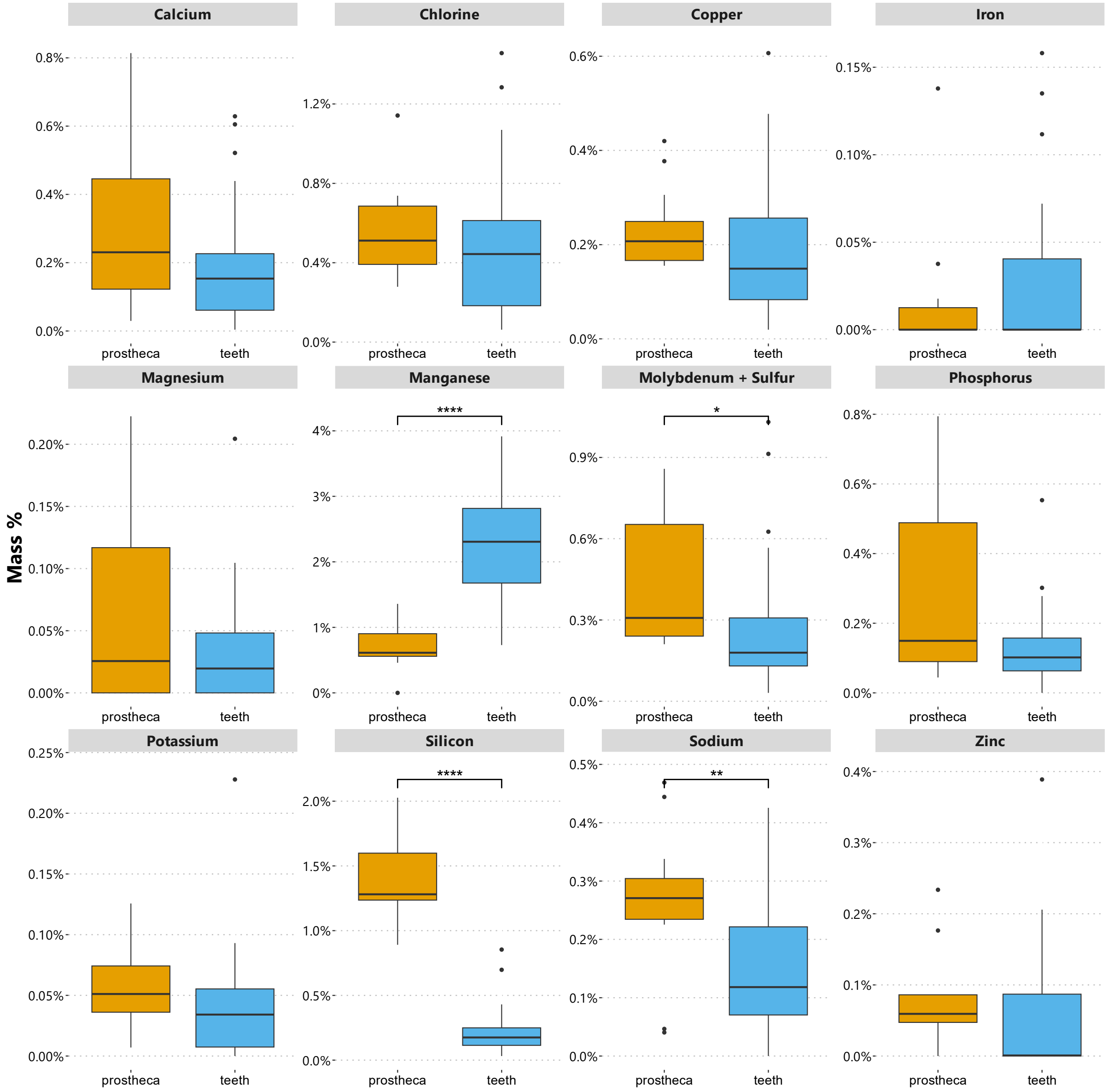

Figure S2. Metal accumulation in the mandibles of *Subcoccinella vigintiquatuorpunctata*. The box plots are showing metal abundance in the prostheca and teeth of *S. vigintiquatuorpunctata*. Center line in each box plots represent the mean value, with standard error bars. Asterisks indicate significant variation in metal accumulation between prostheca and teeth (Wilcoxon test, α=0.05).

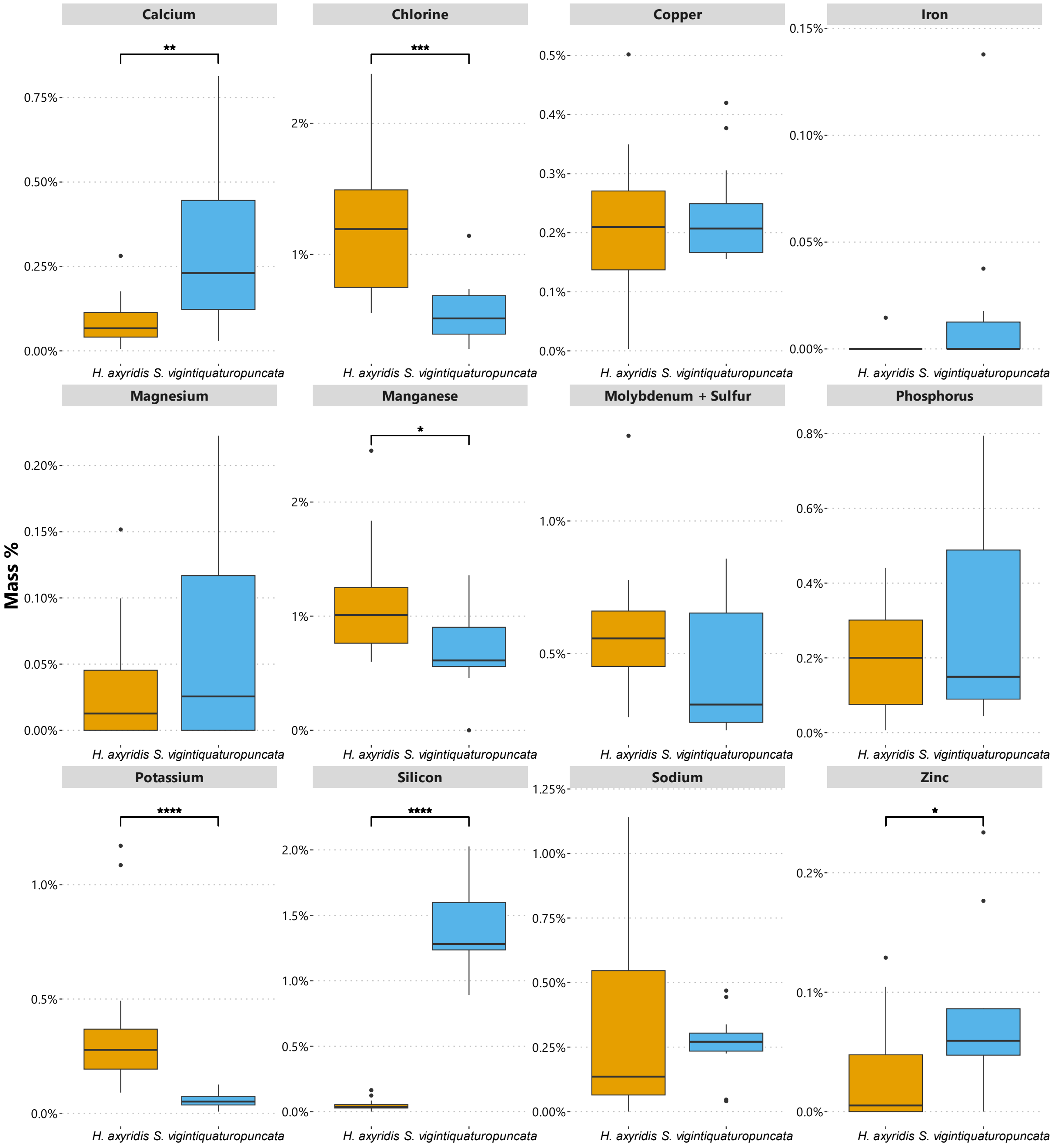

Figure S3. Differences in metal accumulation at the teeth level in *Harmonia axyridis* and *Subcoccinella vigintiquatuorpunctata.* Asterisks indicate significant variation in the metal accumulation between species (Wilcoxon test, α=0.05).

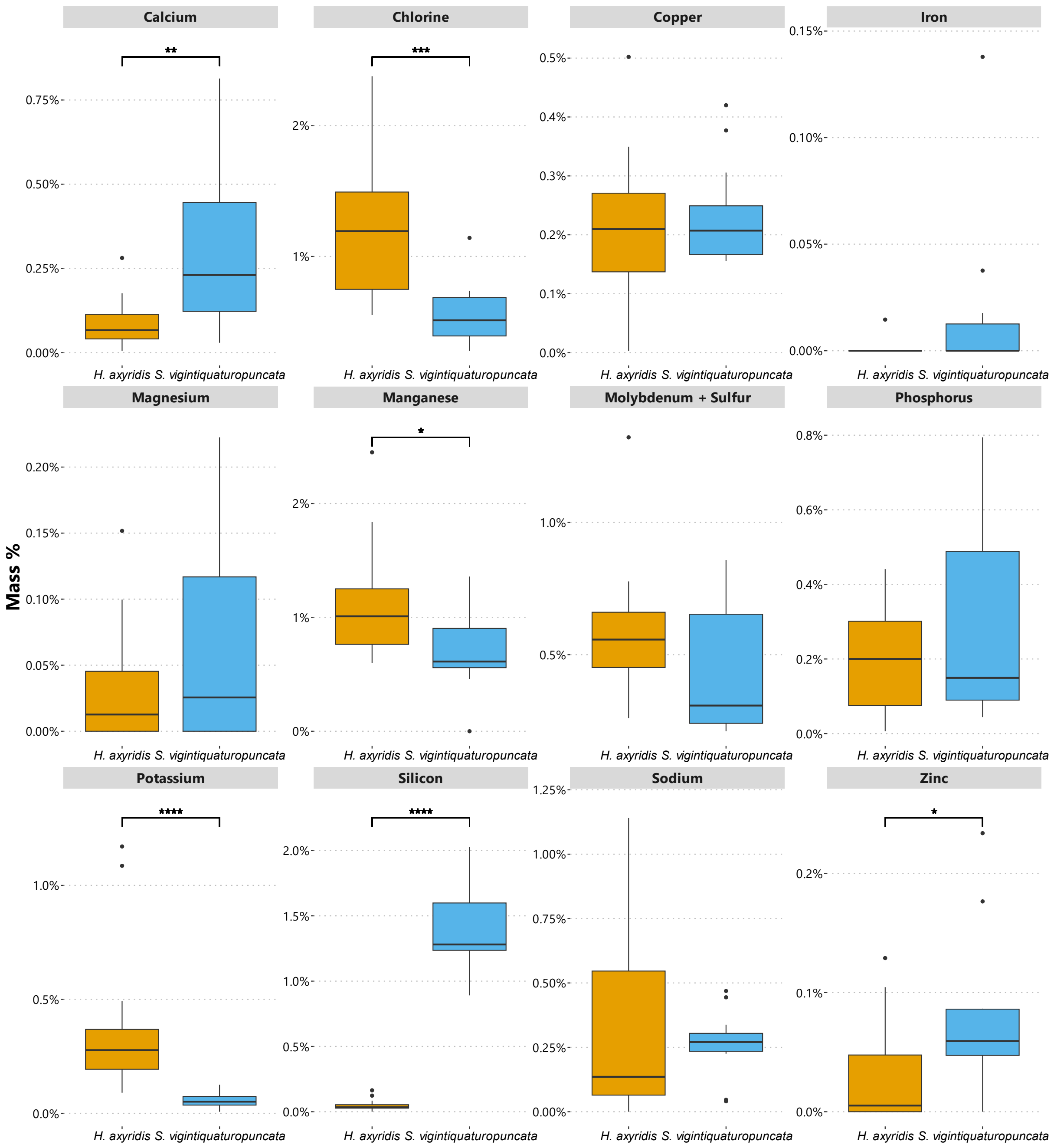

Figure S4. Differences in metal accumulation at the prostheca level in *Harmonia axyridis* and *Subcoccinella vigintiquatuorpunctata.* Asterisks indicate significant variation in the metal accumulation between species (Wilcoxon test, α=0.05).
